# Cognitive performance of adult patients with SMA before and after treatment initiation with nusinersen

**DOI:** 10.1186/s12883-023-03261-z

**Published:** 2023-06-06

**Authors:** Maximilian Vidovic, Maren Freigang, Elisa Aust, Katharina Linse, Daniel Petzold, René Günther

**Affiliations:** 1 Department of Neurology, University Hospital Carl Gustav Carus, Technische Universität Dresden, Dresden, Germany; 2 German Center for Neurodegenerative Diseases, Dresden, Dresden, Germany

**Keywords:** Spinal muscular atrophy (SMA), Cognition, Cognitive performance, Edinburgh cognitive and behavioral ALS screen (ECAS), Nusinersen

## Abstract

**Background:**

Spinal muscular atrophy (SMA) is a genetic neuromuscular disease caused by mutations of the *SMN1* gene. Deficient SMN protein causes irreversible degeneration of alpha motor neurons characterized by progressive muscle weakness and atrophy. Considering that SMA is a multi-systemic disorder and SMN protein was found to be expressed in cortical structures, the cognitive profile of adult patients with SMA has recently been of particular interest. With nusinersen, a novel, disease-modifying drug has been established, but its effects on neuropsychological functions have not been validated yet. Aim of this study was to investigate the cognitive profile of adult patients with SMA during treatment initiation with nusinersen and to reveal improvement or deterioration in cognitive performance.

**Methods:**

This monocentric longitudinal study included 23 patients with SMA type 2 and 3. All patients were assessed with the Edinburgh Cognitive and Behavioral ALS Screen (ECAS) before and after 14 months of treatment initiation with nusinersen. Additionally, motor function was evaluated by Hammersmith Functional Motor Scale Expanded (HFMSE), Revised Upper Limb Module (RULM) and Amyotrophic Lateral Sclerosis Functional Rating Scale Revised (ALSFRS-R).

**Results:**

Of the treatment-naive patients, only three were below the age- and education-matched cut-off for cognitive impairment in the ECAS total score. Significant differences between SMA type 2 and 3 were only detected in the domain of Language. After 14 months of treatment, patients showed significant improvement of absolute scores in all three ALS-specific domains, in the non-ALS-specific domain of Memory, in both subscores and in the ECAS total score. No associations were detected between cognitive and functional outcome measures.

**Conclusions:**

In some adult patients with SMA abnormal cognitive performance in ALS-specific functions of the ECAS was evident. However, the presented results suggest no clinically significant cognitive changes during the observed treatment period with nusinersen.

**Supplementary Information:**

The online version contains supplementary material available at 10.1186/s12883-023-03261-z.

## Introduction

5q-associated spinal muscular atrophy (SMA) is a neurodegenerative autosomal recessive disorder caused by homozygous deletion or mutation in the survival motor neuron 1 gene (*SMN1*). It is characterized by degeneration of alpha motor neurons in the spinal cord leading to progressive atrophy and weakness of proximal limb muscles predominantly. Regarding age of onset and achieved motor milestones, the disease is classified into four different phenotypes [1, 2]. SMA type 1 (Werdnig-Hoffmann disease) is the most severe form with onset before 6 months of age, severe muscle weakness including bulbar involvement, lack of motor development, inability to sit upright and respiratory failure with poor life expectancy [3]. SMA type 2 is referred to as the intermediate form of SMA and presents predominant progressive proximal leg weakness. These patients are able to sit at some point but are unable to stand or walk independently. Children and adults with SMA type 3 (Kugelberg-Welander disease) achieve the ability to walk independently during development and show sparse comorbidities such as scoliosis and respiratory muscle weakness of varying severity [2]. Bulbar motor dysfunction may be present in SMA type 2 and 3, while patients with SMA type 2 are more likely to be affected [4]. SMA type 4 represents the mildest form with late onset in adulthood, comprising less than 5% of all SMA cases [2].

Considering proven vulnerability of additional cell and tissue types to reduced levels of SMN protein, SMA has lately been discussed as a multi-systemic disorder [5]. However, patient-reported non-motor symptoms appear to be rare in adult patients [6].

Although the cognitive profile of patients with SMA has not been extensively studied, it has raised particular interest as SMN protein was found to be expressed in regions of the forebrain [7]. Published studies on cognitive abilities showed no evidence of cognitive impairment in children with SMA types 2 and 3, whereas children with SMA type 1 are more likely to be affected. This particularly concerns attention and executive function [8]. Recent studies focused on cognitive profile of adult patients with SMA. Compared to amyotrophic lateral sclerosis (ALS), a fatal neurodegenerative disease affecting upper and lower motor neurons, adult patients with SMA showed evidence of better performance in cognitive domains of memory, language and executive function [9]. A study with healthy controls showed that cognitive abilities were within normal range in adult patients with SMA. However, executive function inversely correlated with motor function, suggesting a compensation to physical restrictions in SMA [10]. In contrast, lower IQ index scores were found for working memory and perceptual reasoning in patients with SMA type 2 than in general population, encouraging SMA as a multi-systemic disease and disapproving the hypothesis of improved cognitive skills as a compensation for physical impairment [11].

Above-mentioned studies on cognitive performance of adult patients with SMA were done cross-sectionally with all patients being under treatment with the antisense oligonucleotide nusinersen. Though very low concentrations of the intrathecally applied drug were measured in brain cells [12], a possible nusinersen effect on cognitive function remains unclear and has not been clinically evaluated yet.

This longitudinal study aimed to investigate cognitive performance of adult patients with SMA before and following treatment initiation with nusinersen and to draw attention to possible positive or adverse effects on the cognitive profile. Additional correlation analyses of cognitive and motor function scores were conducted to gain complementary knowledge about the relation between cognitive and motor abilities during treatment.

## Materials and methods

### Study design, patients, physical and cognitive examination

In a monocentric, prospective, longitudinal study, patients with SMA type 2 (n = 10) and SMA type 3 (n = 15) were enrolled between 2017 and 2020 at the University hospital Dresden, Germany. The local ethics committee approved the study and all patients signed written informed consent. Disease-specific mutations of the *SMN1* gene were genetically confirmed in all patients. One patient with SMA type 3 discontinued treatment after receiving the four loading doses and did not participate in follow-up visits. Another patient with SMA type 2, who received initial treatment (up to ten months) in the department of neuropediatrics, did not undergo neurocognitive testing at baseline visit.

A total of 23 adult patients with SMA were included in the final analysis. All patients were older than 18 years at the time of inclusion. One patient had pre-diagnosed depression and underwent a long-time antidepressant therapy without current signs of relapse. The other patients had no relevant psychiatric or cognitive comorbidities in medical history or at the time of the investigation.

Clinical and neuropsychological examination was conducted in two visits. The baseline visit (V_0_) was held just before the first loading dose of nusinersen. The follow-up visit (V_1_) was done 14 months after V_0_, constituting one year after completed treatment initiation.

Motor function was evaluated using Hammersmith Functional Motor Scale Expanded (HFMSE), Revised Upper Limb Module (RULM) and Amyotrophic Lateral Sclerosis Functional Rating Scale Revised (ALSFRS-R).

The neurocognitive profile was assessed using the German version of the Edinburgh Cognitive and Behavioral ALS Screen (ECAS). It was originally designed to identify cognitive impairment in patients with ALS and comprises three ALS-specific task domains (Language, Verbal Fluency and Executive Functions) and two non-ALS-specific task domains (Memory and Visuospatial functions) [13]. The ratings of these domains result in an ALS-specific and a non-ALS-specific subscore, which were separately analyzed in addition to the ECAS total score. Because of their influence on cognition, age- and education-matched cut-off scores were applied to determine cognitive impairment [14]. The ECAS also includes a questionnaire to address behavioral changes that, however, was not applied in this study. Although not specifically directed to patients with SMA, it has been used frequently and was claimed to be an appropriate neuropsychological screening due to taking motor disabilities into account [9, 10].

### Statistical analysis

Sample characteristics are displayed as mean (± standard deviation), minimum and maximum (range) or frequency (percentage) for both SMA types and total study population.

Shapiro-Wilk-test was used to test for normal distribution of continuous variables. Group comparisons were performed with either one-sample t-test for normally distributed data or with Mann-Whitney U-test for data for which normal distribution could not be assumed. For paired analysis of repeated measures, Wilcoxon signed-rank test was performed. Fisher’s exact test was used to analyze the differences between the groups with regard to the proportion of ECAS scores below the cut-off indicating cognitive impairment. Associations between ECAS outcome scores and number of *SMN2* copies were determined by calculating the Spearman’s correlation coefficient. Associations between ECAS outcome scores and levels of motor rating scores were investigated by partial correlation analyses with age and education years as covariates. All tests were two-sided, with a *p* value < 0.05 considered as statistically significant. Conclusively, effect sizes were reported considering *r* = 0.1 as a weak, *r* = 0.3 as a moderate and *r* = 0.5 as a strong effect [15].

Statistical analysis was performed using SPSS software (IBM Corp., Version 27.0. Armonk, NY) and data visualization was done using GraphPad Prism Software version 9.3 (GraphPad Software, La Jolla California USA).

## Results

### Demographic characteristics

Mean baseline age of all included patients was 38.1 (± 11.4) years. Patients with SMA type 2 were significantly younger compared to those with SMA type 3 (*p* = 0.033). No significant differences between both types were found for sex, ambulatory status, disease duration and education level (Table 1).

**Table 1:** Demographic and disease characteristics

	Total (n = 23)	SMA type 2 (n = 9)	SMA type 3 (n = 14)	SMA type 2 vs. SMA type 3
Age at baseline, years				**t(21) = − 2.29;*p*** **= 0.033**^**1**^; ***d*** **= 0.98**
Mean ± SD Median (Range)	38.1 ± 11.4 35.0 (18–57)	31.9 ± 10.8 30.0 (18–54)	42.1 ± 10.2 42.5 (22–57)	
Sex, n (%)				*p* = 0.669^2^; OR = 2.00
Female	10 (43.5)	3 (33.3)	7 (50.0)	
Male	13 (56.5)	6 (66.7)	7 (50.0)	
*SMN2* copy number, n (%)				***U*** **= 28.5;*Z*= − 2.464;*p*** **= 0.028**^**3**^; ***r*** **= 0.51**
2	2 (8.7)	1 (11.1)	1 (7.1)	
3	13 (56.5)	8 (88.9)	5 (35.7)	
4	8 (34.8)	0 (0.0)	8 (57.1)	
Mobility, n (%)				*p* = 0.116^2^; OR = 1.56
Ambulatory	5 (21.7)	0 (0.0)	5 (37.5)	
Non-ambulatory	18 (78.3)	9 (100.0)	9 (64.3)	
Disease duration, years				*U* = 43.5; *Z* = − 1.231; *p* = 0.224^3^; *r* = 0.26
Mean ± SD Median (Range)	34.6 ± 11.1 32.0 (16–54)	31.0 ± 11.0 29.3 (16–54)	36.9 ± 10.8 34.9 (20–49)	
Education level, years				*t*(21) = 1.09; *p* = 0.258^1^; *d* = 0.48
Mean ± SD Median (Range)	13.5 ± 3.1 13.0 (9–19)	14.4 ± 3.6 13.0 (9–19)	12.9 ± 2.7 12.5 (10–18)	

SMA: spinal muscular atrophy; SMN2: survival motor neuron 2 gene; SD: standard deviation; n: number; ^1^calculated by one-sample t-test; ^2^calculated by Fisher’s exact test; ^3^calculated by Mann-Whitney U-test; *p* values < 0.05 considered statistically significant; Cohen’s effect sizes of *r* = 0.10, *r* = 0.30 and *r* = 0.50 as threshold for small, medium and large effects respectively [15]

### Cognitive performance in treatment-naive patients with SMA

**Fig. 1:**
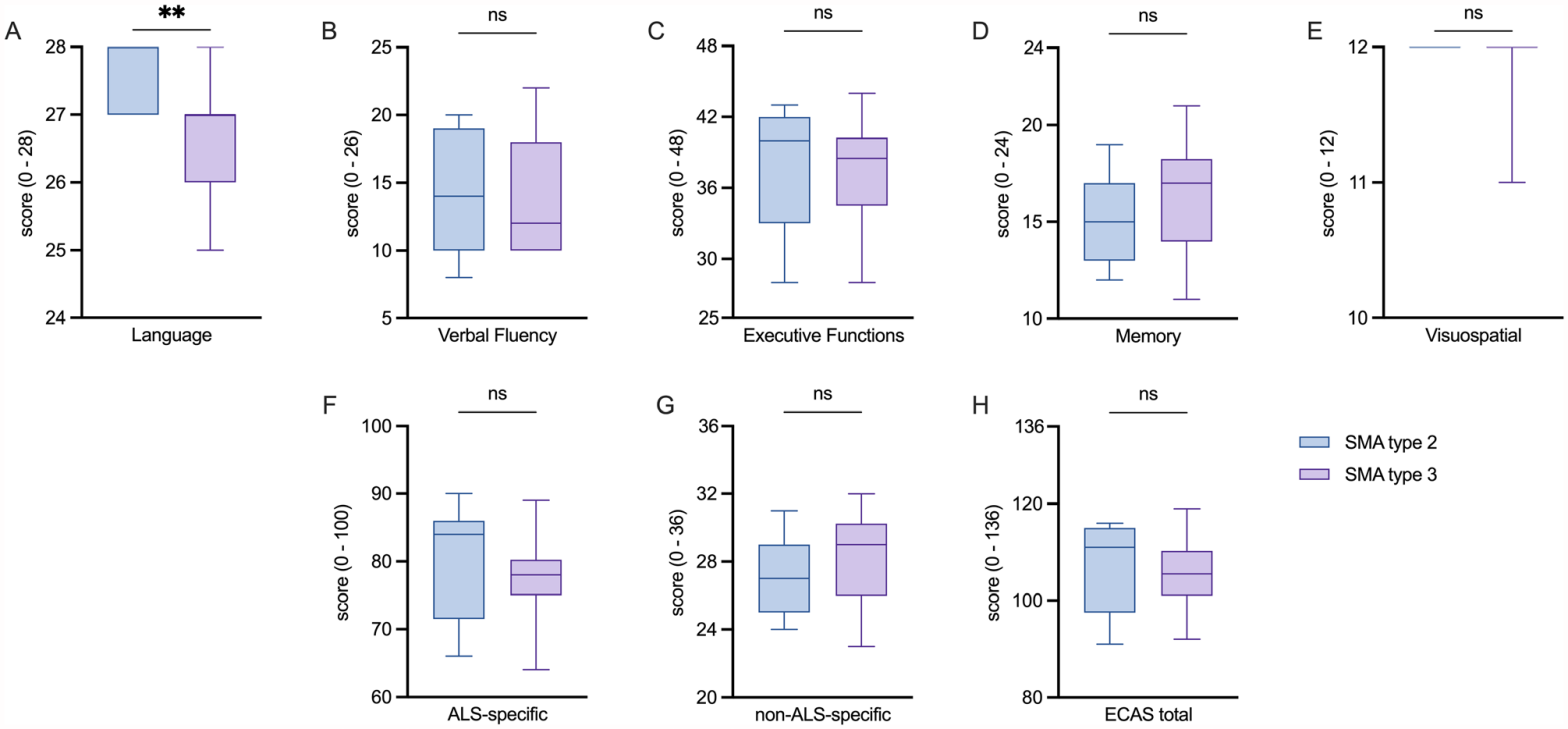
Comparison of ECAS absolute outcome scores of patients with SMA type 2 and 3 before treatment initiation (V_0_) **A, B, C**: Box plots of the ALS-specific domains in patients with SMA type 2 compared to patients with SMA type 3. **D, E**: Box plots of the non-ALS-specific domains in patients with SMA type 2 compared to patients with SMA type 3. **F, G**: Box plots of ECAS subscores in patients with SMA type 2 compared to patients with SMA type 3. **H**: Boxplot of ECAS total score in patients with SMA type 2 compared to patients with SMA type 3. ALS: amyotrophic lateral sclerosis; ECAS: Edinburgh Cognitive and Behavioral ALS Screen; SMA: spinal muscular atrophy. Box plots show median (horizontal line), inter-quartile range (boxes) and scores outside of inter-quartile range (whiskers). Calculated by Mann-Whitney U-test. Significance levels: ** *p* < 0.01; ns: statistically not significant.

At first, absolute ECAS scores in treatment-naive patients with SMA were compared (Table 2; Fig. 1). No significant differences were detected in the absolute ECAS (sub)scores between patients with SMA type 2 and 3, except for the ALS-specific domain of Language. Here, patients with SMA type 2 scored moderately higher (*p* = 0.009).

In patients with SMA type 2, 33.3% scored below cut-off and were thus classified as impaired in the domain of Verbal Fluency and 22.2% in the domain of Executive Functions. 22.2% were classified as impaired regarding the ALS-specific subscore and the ECAS total score, respectively. None of them were below cut-off score in the other domains or the non-ALS-specific subscore.

In patients with SMA type 3, 14.3% showed impairment in the ALS-specific domains of Language and Executive Functions, respectively. 28.6% displayed impairment in the domain of Verbal Fluency and 7.1% in the non-ALS-specific domain of Visuospatial Functions. 50.0% were classified as impaired based on the ALS-specific subscore, whereas none of the patients was based on the non-ALS-specific subscore. In contrast to SMA type 2, only 7.1% of patients with SMA type 3 were classified as impaired using the ECAS total score. However, no significant group differences between treatment-naive patients with SMA type 2 and 3 regarding age- and education-matched cut-off scores for the ECAS (sub)scores were observed (Table 2).

Furthermore, ECAS total scores did not correlate with the number of *SMN2* copies in treatment-naive patients with SMA type 2 (*ρ* = − 0.557, *p* = 0.222) and type 3 (*ρ* = 0.188, *p* = 0.513).

**Table 2:** ECAS outcome scores in treatment-naive patients with SMA

ECAS outcome scores – V_0_ Median (Range)	SMA type 2 (n = 9)	SMA type 3 (n = 14)	SMA type 2 vs. SMA type 3
Language (max. 28)	28.0 (27–28)	27.0 (25–28)	***U*** **= 22.5;** ***Z*** **= − 2.728;** ***p*** **= 0.009**^**1**^; ***r*** **= 0.57**
below cut-off, n (%)	0 (0.0)	2 (14.3)	*p* = 0.502^2^; OR = 1.17
Verbal Fluency (max. 24)	14.0 (8–20)	12.0 (10–22)	*U* = 56.5; *Z* = − 0.421; *p* = 0.688^1^; *r* = 0.09
below cut-off, n (%)	3 (33.3)	4 (28.6)	*p* = 0.999^2^; OR = 0.80
Executive Functions (max. 48)	40.0 (28–43)	38.5 (28–44)	*U* = 55.0; *Z* = − 0.506; *p* = 0.643^1^; *r* = 0.11
below cut-off, n (%)	2 (22.2)	2 (14.3)	*p* > 0.999^2^; OR = 0.58
Memory (max. 24)	15.0 (12–19)	17.0 (11–21)	*U* = 42.5; *Z* = − 1.301; *p* = 0.201^1^; *r* = 0.27
below cut-off, n (%)	0 (0.0)	0 (0.0)	*p* > 0.999^2^
Visuospatial (max. 12)	12.0 (12)	12.0 (11–12)	*U* = 54.0; *Z* = − 1.161; *p* = 0.600^1^; *r* = 0.24
below cut-off, n (%)	0 (0.0)	1 (7.1)	*p* > 0.999^2^; OR = 1.01
ALS-specific (max. 100)	84.0 (66–90)	78.0 (64–89)	*U* = 51.0; *Z* = − 0.757; *p* = 0.477^1^; *r* = 0.16
below cut-off, n (%)	2 (22.2)	7 (50.0)	*p* > 0.999^2^; OR = 1.25
Non-ALS-specific (max. 36)	27.0 (24–31)	29.0 (23–32)	*U* = 43.0; *Z* = − 1.270; *p* = 0.224^1^; *r* = 0.26
below cut-off, n (%)	0 (0.0)	0 (0.0)	*p* > 0.999^2^
ECAS total (max. 136)	111.0 (91–116)	105.5 (92–119)	*U* = 56.0; *Z* = − 0.442; *p* = 0.688^1^; *r* = 0.09
below cut-off, n (%)	2 (22.2)	1 (7.1)	*p* = 0.538^2^; OR = 0.27

ALS: amyotrophic lateral sclerosis; ECAS: Edinburgh Cognitive and Behavioral ALS Screen; SMA: spinal muscular atrophy; SD: standard deviation; n: number; OR: odds ratio; V_0_: Baseline visit before treatment initiation; ^1^calculated by Mann-Whitney U-test; ^2^calculated by Fisher’s exact test; *p* values < 0.05 considered statistically significant and are marked bold; Cohen’s effect sizes of *r* = 0.10, *r* = 0.30 and *r* = 0.50 as threshold for small, medium and large effects respectively [15]

To investigate if ECAS scores showed any association with motor impairment, partial correlation analysis was carried out for each motor function assessment with age and educational level as covariates. In all three applied assessments, no significant correlations were detected between cognitive and functional outcome measures before treatment initiation with nusinersen (Supplementary Material, Table S1).

### Changes in cognitive performance during 14 months of nusinersen treatment

**Fig. 2:**
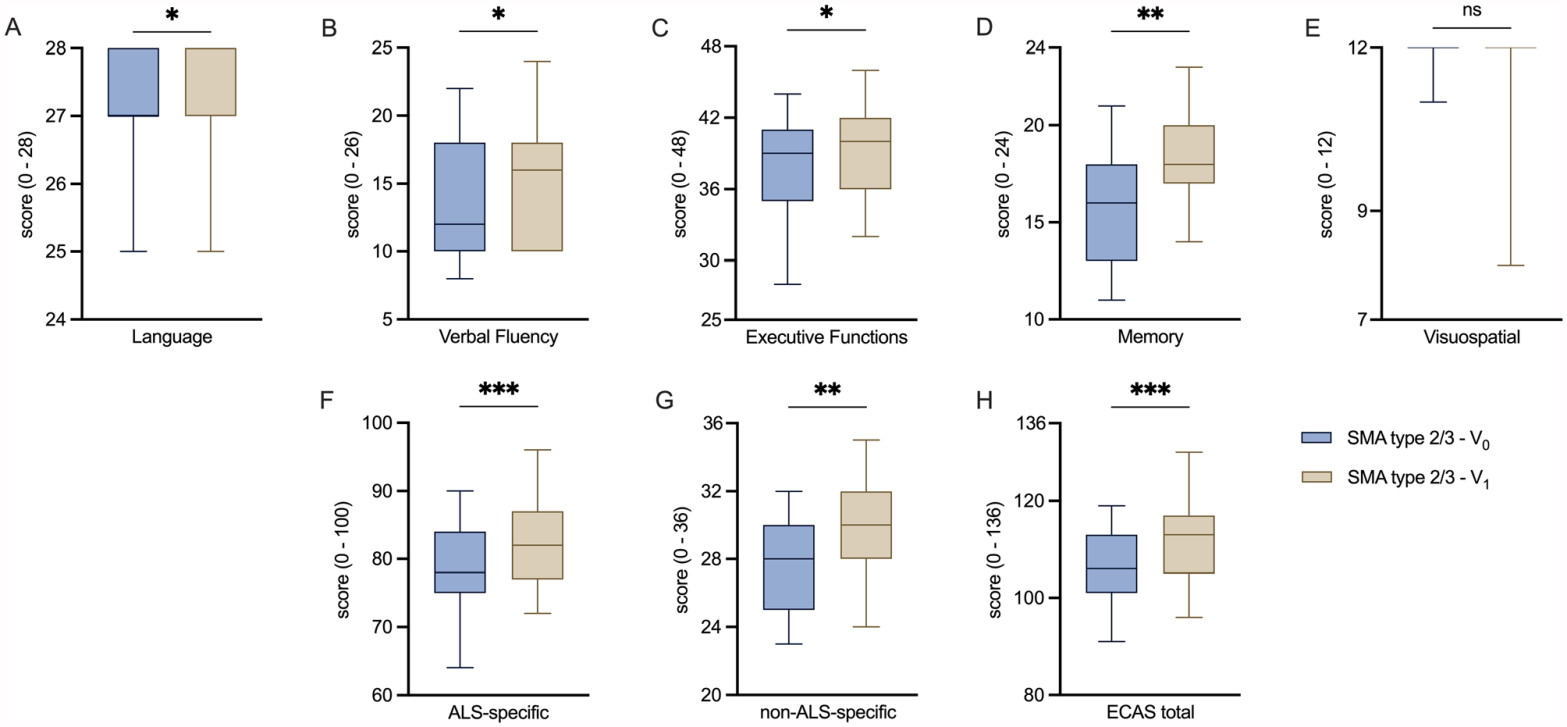
Comparison of ECAS absolute scores before and after treatment initiation **A, B, C**: Box plots of the ALS-specific domains before and after treatment initiation with nusinersen in SMA cohort. **D, E**: Box plots of the non-ALS-specific domains before and after treatment initiation with nusinersen in SMA cohort. **F, G**: Box plots of ECAS subscores before and after treatment initiation with nusinersen in SMA cohort. **H**: Box plots of ECAS total score before and after treatment initiation with nusinersen in SMA cohort. ALS: Amyotrophic lateral sclerosis; ECAS: Edinburgh Cognitive and Behavioral ALS Screen; SMA: Spinal muscular atrophy. V_0_: Baseline visit before treatment initiation; V_1_: Follow-up visit 14 months after treatment initiation. Box plots show median (horizontal line), inter-quartile range (boxes) and scores outside of inter-quartile range (whiskers). Calculated by Wilcoxon signed-rank test. Significance levels: * *p* < 0.05; ** *p* < 0.01; *** *p* < 0.001; ns: statistically not significant

**Fig. 3:**
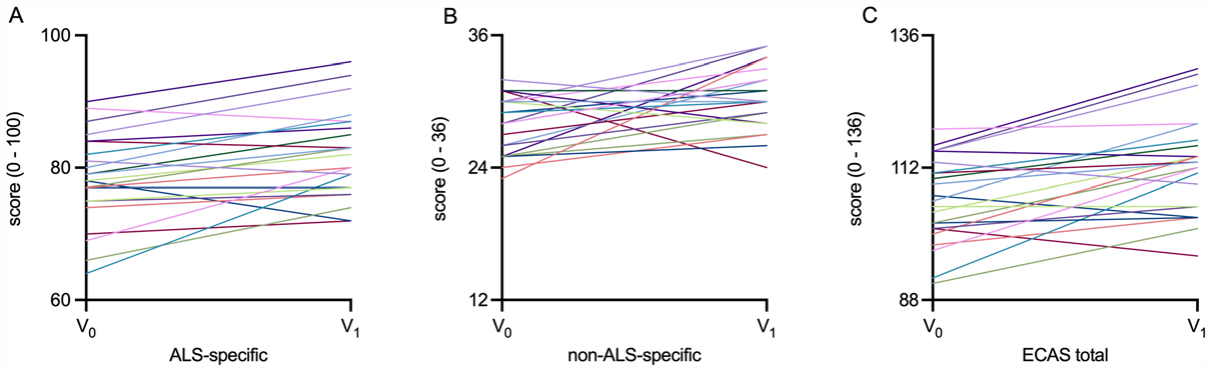
Spaghetti plots for longitudinal changes in ECAS of SMA cohort Spaghetti plots for **A**: ALS-specific subscore, **B**: non-ALS-specific subscore, **C**: ECAS total score. ALS: amyotrophic lateral sclerosis; ECAS: Edinburgh Cognitive and Behavioral ALS Screen; SMA: spinal muscular atrophy. V_0_: Baseline visit before treatment initiation; V_1_: Follow-up visit 14 months after treatment initiation. Each line represents an individual patient

A longitudinal analysis of absolute and cut-off ECAS scores was implemented for the total SMA cohort, comparing the cognitive performance before and after 14 months of nusinersen treatment (Table 3; Figs. 2 and 3).

The patients showed significant improvement of absolute scores in all three ALS-specific domains (Language: *p* = 0.019; Verbal Fluency: *p* = 0.028; Executive Function: *p* = 0.014), in the non-ALS-specific domain of Memory (*p* = 0.003) as well as in both subscores (ALS-specific: *p* = 0.001; non-ALS-specific: *p* = 0.006) and in the ECAS total score (*p* < 0.001).

After 14 months, 34.8% had impairment as assessed by the ALS-specific subscore, whereas no patient was classified as impaired in the non-ALS-specific subscore. None of the patients scored below cut-off values in the ECAS total score. Non-ALS-specific Visuospatial Functions represented the most affected domain with impairment in 17.4% of the patients, followed by 13.0% in Verbal Fluency and 4.3% in Language and Executive Functions, respectively.

**Table 3:** ECAS outcome scores of patients with SMA type 2 and 3 before and after treatment initiation

ECAS outcome scores Median (Range)	V_0_ – SMA type 2/3 (n = 23)	V_1_ - SMA type 2/3 (n = 23)	V_0_ vs. V_1_
Language (max. 28)	27.0 (25–28)	28.0 (25–28)	***Z*** **= − 2.352;** ***p*** **= 0.019** ^**1**^; ***r*** **= 0.49**
below cut-off, n (%)	2 (8.7)	1 (4.3)	*p* > 0.999^2^; OR = 0.48
Verbal Fluency (max. 24)	12.0 (8–22)	16.0 (10–24)	***Z*= − 2.199;*p*** **= 0.028**^**1**^; ***r*** **= 0.46**
below cut-off, n (%)	7 (30.4)	3 (13.0)	*p* = 0.284^2^; OR = 0.34
Executive Functions (max. 48)	39.0 (28–44)	40.0 (32–46)	***Z*= − 2.469;*p*** **= 0.014**^**1**^; ***r*** **= 0.51**
below cut-off, n (%)	4 (17.4)	1 (4.3)	*p* = 0.346^2^; OR = 0.22
Memory (max. 24)	16.0 (11–21)	18.0 (14–23)	***Z*= − 2.979;*p*** **= 0.003**^**1**^; ***r*** **= 0.62**
below cut-off, n (%)	0 (0.0)	0 (0.0)	*p* > 0.999^2^
Visuospatial (max. 12)	12.0 (11–12)	12.0 (8–12)	*Z* = − 1.511; *p* = 0.131^1^; *r* = 0.81
below cut-off, n (%)	1 (4.3)	4 (17.4)	*p* = 0.346^2^; OR = 4.63
ALS-specific (max. 100)	78.0 (64–90)	82.0 (72–96)	***Z*= − 3.241;*p*** **= 0.001**^**1**^; ***r*** **= 0.68**
below cut-off, n (%)	11 (47.8)	8 (34.8)	*p* = 0.550^2^; OR = 0.58
Non-ALS-specific (max. 36)	28.0 (23–32)	30.0 (24–35)	***Z*= − 2.754;*p*** **= 0.006**^**1**^; ***r*** **= 0.57**
below cut-off, n (%)	0 (0.0)	0 (0.0)	*p* > 0.999^2^
ECAS total (max. 136)	106.0 (91–119)	113.0 (96–130)	***Z*= − 3.318;*p*** **< 0.001**^**1**^; ***r*** **= 0.69**
below cut-off, n (%)	3 (13.0)	0 (0.0)	*p* = 0.233^2^; OR = 0.00

ALS: amyotrophic lateral sclerosis; ECAS: Edinburgh Cognitive and Behavioral ALS Screen; n: number; SMA: spinal muscular atrophy; SD: standard deviation; OR: odds ratio; V_0_: Baseline visit before treatment initiation; V_1_: Follow-up visit 14 months after treatment initiation; ^1^calculated by Wilcoxon signed-rank test for absolute ECAS scores in V_0_ and V_1_; ^2^calculated by Fisher’s exact test (differences between the groups with regard to the proportion of ECAS scores below the cut-off indicating cognitive impairment in V_0_ and V_1_); *p* values < 0.05 considered statistically significant and are marked bold; Cohen’s effect sizes of *r* = 0.10, *r* = 0.30, and *r* = 0.50 as thresholds for small, medium and large effects respectively [15]

### Motor functions and cognitive performance during 14 months of nusinersen treatment

**Fig. 4:**
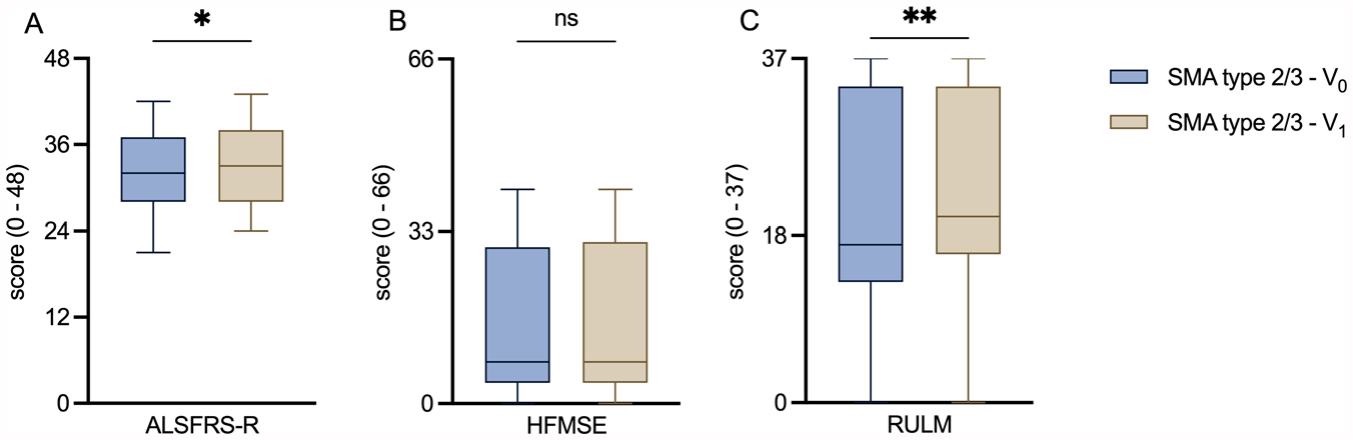
Comparison of motor outcome scores before and after treatment initiation **A, B, C**: Box plots of the motor outcome scores before and after treatment initiation with nusinersen in SMA cohort. ALSFRS-R: ALS Functional Rating Scale Revised; HFMSE: Hammersmith Functional Motor Scale Expanded; RULM: Revised Upper Limb Module; SMA: Spinal muscular atrophy V_0_: Baseline visit before treatment initiation; V_1_: Follow-up visit 14 months after treatment initiation. Box plots show median (horizontal line), inter-quartile range (boxes) and scores outside of inter-quartile range (whiskers). Calculated by Wilcoxon signed-rank test. Significance levels: * *p* < 0.05; ** *p* < 0.01; ns: statistically not significant

Considering the outcome scores on motor functions, our cohort already showed a slight improvement in the ALSFRS-R (median score before treatment initiation: 32.0 points, median score after treatment initiation: 33.0 points; *p* = 0.015) and the RULM (median score before treatment initiation: 17.0 points, median score after treatment initiation: 20.0 points; *p* = 0.005). No significant improvement was observed in the HFMSE (Fig. 4).

Absolute ECAS outcome scores did not correlate with functional motor scores after 14 months of nusinersen treatment (Supplementary Material, Table S1).

**Fig. 5:**
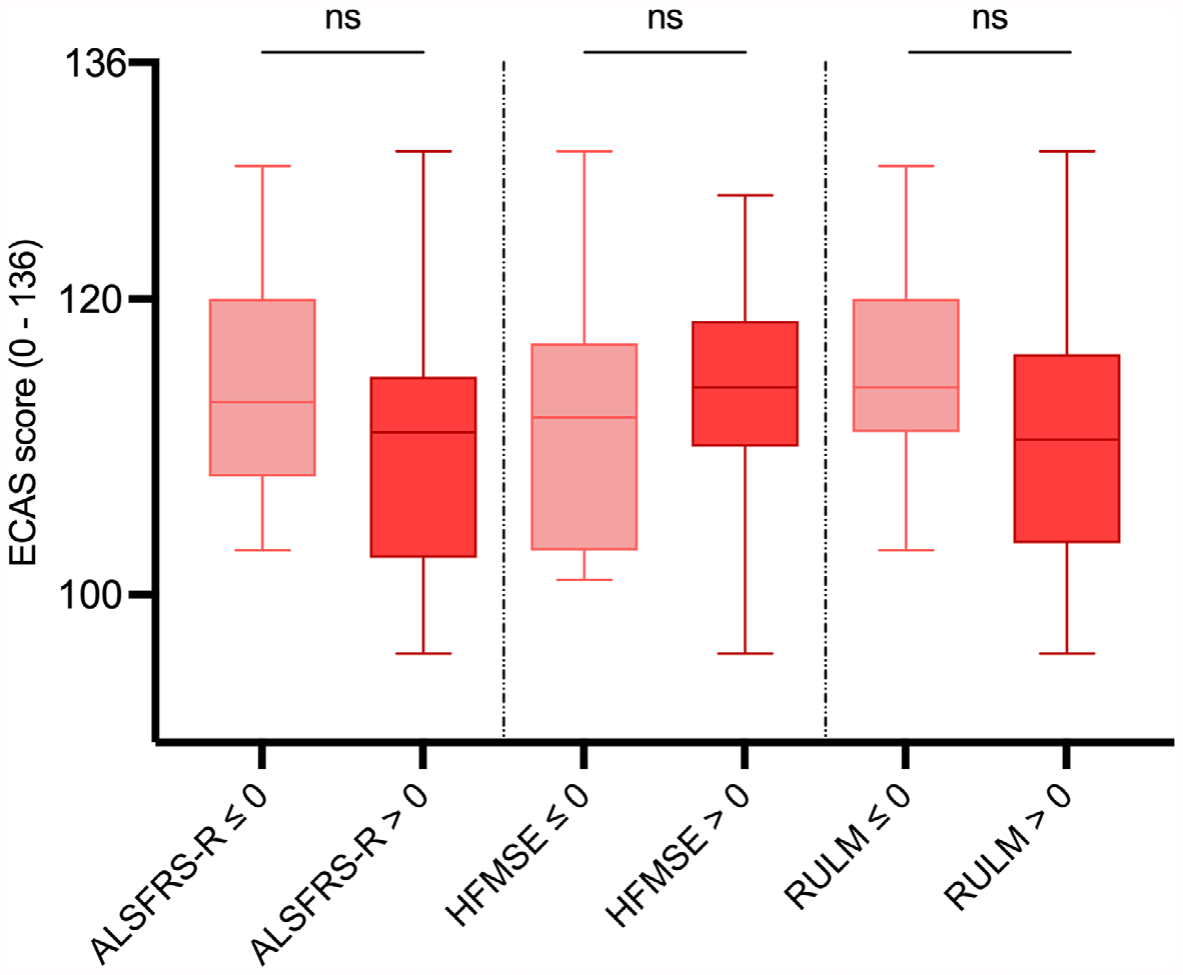
Comparison of ECAS total scores between SMA type 2/3 patients with improvement (score change > 0) and without improvement (score change ≤ 0) in motor outcome scores ALSFRS-R: ALS Functional Rating Scale Revised; ECAS: Edinburgh Cognitive and Behavioral ALS Screen; HFMSE: Hammersmith Functional Motor Scale Expanded; RULM: Revised Upper Limb Module; ns: statistically not significant; Box plots show median (horizontal line), inter-quartile range (boxes) and scores outside of inter-quartile range (whiskers). Calculated by Mann-Whitney U-test

We finally analyzed, if changes in ECAS scores were associated with changes in the motor scores, comparing ECAS scores of patients showing improvement in motor scores (score change > 0) with ECAS scores of patients without improvement (score change ≤ 0). No significant associations were found between changes in ECAS total score and changes in any of the presented motor outcome scores (Fig. 5).

## Discussion

SMA is an inherited neurodegenerative disease which is primarily accompanied by progressive motor impairment, but recent evidence suggests a multi-systemic disease. To date, research has been concentrated on pathophysiological mechanisms and clinical phenotypes regarding motor impairment, while cognitive performance in patients with SMA has merely played a minor role [16].

In addition, previous data mainly focused on the cognitive profile in children with SMA. These studies predominantly disclosed normal or even enhanced cognitive performance [17–19]. This may be explained as cognitive adaptation to their physical disability, reallocating cognitive resources no longer needed for motor tasks [19, 20] as well as extensive social interaction to caregivers.

Cognitive performance in adult patients with SMA has lately become subject of scientific interest. Recent comparative studies with ALS patients and controls have reported no considerable cognitive impairment in adult patients with SMA [9, 10]. However, a trend towards lower cognitive performance in comparison to healthy controls was supposed, emphasizing SMA as a multi-systemic disorder [11]. Yet, there is little information about the effect of nusinersen on neuropsychological functions.

With nusinersen a novel disease-modifying treatment has been approved, showing improvement in motor function within 14 months of treatment not only in children but also in adults [21]. By intrathecal application the drug distributes within the cerebrospinal fluid and is taken up into glial cells throughout the CNS in the spinal cord and the brainstem as well as in rostral brain regions such as the frontal and temporal cortex, thalamus, cerebellum and hippocampus [22]. Although these pharmacological studies revealed concentrations of nusinersen in the cerebrum [22], its effect on higher cortical functions such as cognition has not been validated in comparative studies yet. Aim of this study was to investigate the cognitive profile of adult patients with SMA during treatment initiation with nusinersen and to reveal possible favoring or adverse changes of their cognitive performance.

Currently, there is no validated cognitive assessment tool exclusively developed for SMA. Intellectual assessments, using instruments as the Wechsler or McCarthy Intelligence Scale, are commonly employed to evaluate cognitive abilities of children with SMA [8]. However, progressive motor dysfunction is not incorporated in these assessment tools. Although the ECAS is specifically conceptualized for ALS, it takes motor disabilities into account and may also provide relevant insights into cognitive and behavioral changes in other motor neuron diseases, such as SMA.

Our study revealed no significant differences between patients with SMA type 2 and 3 in the ECAS absolute scores and proportions according to age- and education-matched cut-off scores at baseline, except for the domain of Language in which SMA type 3 showed significantly lower mean scores.

Noticeable, some patients showed impairments in the ALS-specific domains and the ALS-specific subscore, while no impairment was observed in the non-ALS-specific subscore. These findings are mainly consistent with previous observations, suggesting a possible impact of SMA on cognition, which is comparable to cognitive involvement in ALS. However, in contrast to previous findings [9], we found a different pattern of performance in the ECAS. In our cohort of patients with SMA, the most frequent cognitive impairment was found in the domain of Verbal Fluency.

Proportions of scores below age- and education-matched cut-off for ALS-specific domains and ALS-specific subscore were still evident in the follow up assessment, reducing the probability of random finding. In support of these clinical findings, it would be of particular interest to know whether associated structural changes may also be detected in brain imaging.

After 14 months of treatment with nusinersen, patients with SMA showed significantly higher absolute scores in each ECAS domain (except Visuospatial Functions) as well as higher subscores and a higher ECAS total score compared to baseline assessment. Regarding data of healthy controls, improvements in ECAS were reported and interpreted as a casual practice effect [23]. This may be in accordance with previous methodical findings interpreting ≥ 8, ≥4, or ≥ 9 point changes as significant changes in ALS-specific, non-ALS-specific, or ECAS total score, respectively [24].

Based on these recommendations, no clinically significant changes after 14 months of nusinersen treatment were found in our SMA cohort and observed improvements could rather be explained by a practice effect [23]. Based on our findings we may additionally conclude that nusinersen initiation treatment does not have a negative impact on cognition either. Furthermore, no significant associations between motor functions and cognitive profile were found. Thus, improvement in physical abilities was not accompanied by enhanced or worsened cognitive functions.

A major limitation of the study is the lack of a treatment-naive control group, the relatively small sample size and short observation period. Data interpretation may also be limited because ECAS has not been validated for SMA yet and alternate ECAS versions were not administered in favor of better test-retest reliability in our study.

## Conclusion

Along with recent studies, some adult patients with SMA showed abnormal performance in ALS-specific domains of the ECAS. During observed treatment period with nusinersen, no clinically relevant changes in performance on the cognitive screen of the ECAS were detected.

## Electronic supplementary material

Below is the link to the electronic supplementary material.

Supplementary Material 1

## Data Availability

The datasets used and/or analyzed during the current investigation are available upon reasonable request from the corresponding author.

## Abbreviations

ALS: Amyotrophic lateral sclerosis
ALSFRS-R: ALS Functional Rating Scale Revised
ECAS: Edinburgh Cognitive and Behavioral ALS Screen
HFMSE: Hammersmith Functional Motor Scale Expanded
RULM: Revised Upper Limb Module
SMA: Spinal muscular atrophy
SMN: Survival Motor Neuron
