# Correction: Cognitive performance of adult patients with SMA before and after treatment initiation with nusinersen

**DOI:** 10.1186/s12883-023-03289-1

**Published:** 2023-06-22

**Authors:** Maximilian Vidovic, Maren Freigang, Elisa Aust, Katharina Linse, Daniel Petzold, René Günther

**Affiliations:** 1 Department of Neurology, University Hospital Carl Gustav Carus, Technische Universität Dresden, Dresden, Germany; 2 German Center for Neurodegenerative Diseases, Dresden, Dresden Germany

**Correction: BMC Neurol 23, 216 (2023)**

**https://doi.org/10.1186/s12883-023-03261-z**

Following publication of the original article [1], the authors reported formal errors in the Figs. 1, 2, 3, 4 and 5. The axis labeling was inconsistent and figures were in low resolution. Also, reference #6 (Günther R, Wurster CD, Cordts I, Koch JC, Kamm C, Petzold D, et al. Patient-Reported Prevalence of Non-motor Symptoms Is Low in Adult Patients Suffering From 5q Spinal Muscular Atrophy. Front Neurol. 2019;10:1098. https://doi.org/10.3389/fneur.2019.01098) was incomplete and lacked DOI.

The correct figures are presented below.

**Fig. 1:**
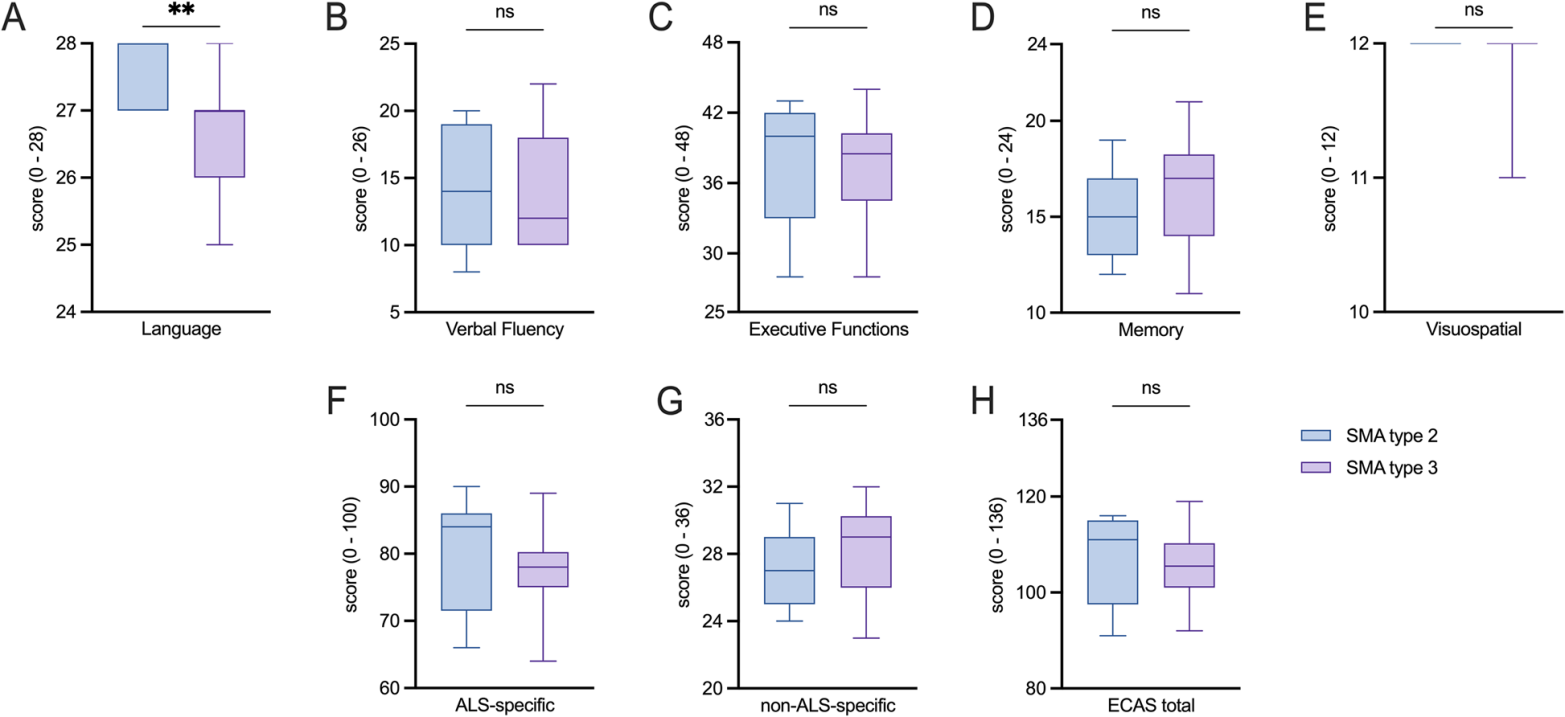
Comparison of ECAS absolute outcome scores of patients with SMA type 2 and 3 before treatment initiation (V0) **A**, **B**, **C**: Box plots of the ALS-specific domains in patients with SMA type 2 compared to patients with SMA type 3. **D**, **E**: Box plots of the non-ALS-specific domains in patients with SMA type 2 compared to patients with SMA type 3. **F**, **G**: Box plots of ECAS subscores in patients with SMA type 2 compared to patients with SMA type 3. **H**: Boxplot of ECAS total score in patients with SMA type 2 compared to patients with SMA type 3. ALS: amyotrophic lateral sclerosis; ECAS: Edinburgh Cognitive and Behavioral ALS Screen; SMA: spinal muscular atrophy. Box plots show median (horizontal line), inter-quartile range (boxes) and scores outside of inter-quartile range (whiskers). Calculated by Mann–Whitney U-test. Significance levels: ** *p* < 0.01; ns: statistically not significant

**Fig. 2:**
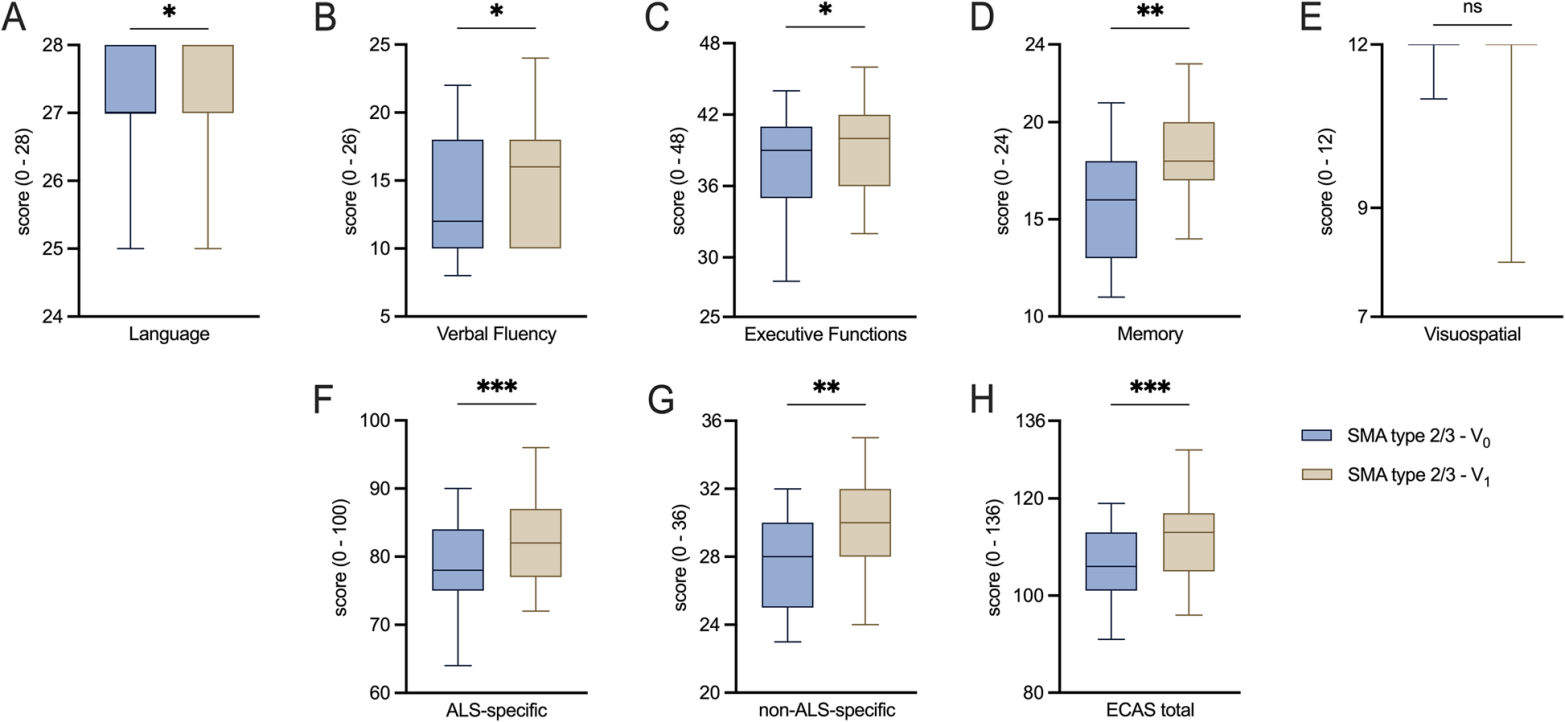
Comparison of ECAS absolute scores before and after treatment initiation **A**, **B**, **C**: Box plots of the ALS-specific domains before and after treatment initiation with nusinersen in SMA cohort. **D**, **E**: Box plots of the non-ALS-specific domains before and after treatment initiation with nusinersen in SMA cohort. **F**, **G**: Box plots of ECAS subscores before and after treatment initiation with nusinersen in SMA cohort. **H**: Box plots of ECAS total score before and after treatment initiation with nusinersen in SMA cohort. ALS: Amyotrophic lateral sclerosis; ECAS: Edinburgh Cognitive and Behavioral ALS Screen; SMA: Spinal muscular atrophy. V0: Baseline visit before treatment initiation; V1: Follow-up visit 14 months after treatment initiation. Box plots show median (horizontal line), inter-quartile range (boxes) and scores outside of inter-quartile range (whiskers). Calculated by Wilcoxon signed-rank test. Significance levels: * *p* < 0.05; ** *p* < 0.01; *** *p* < 0.001; ns: statistically not significant

**Fig. 3:**
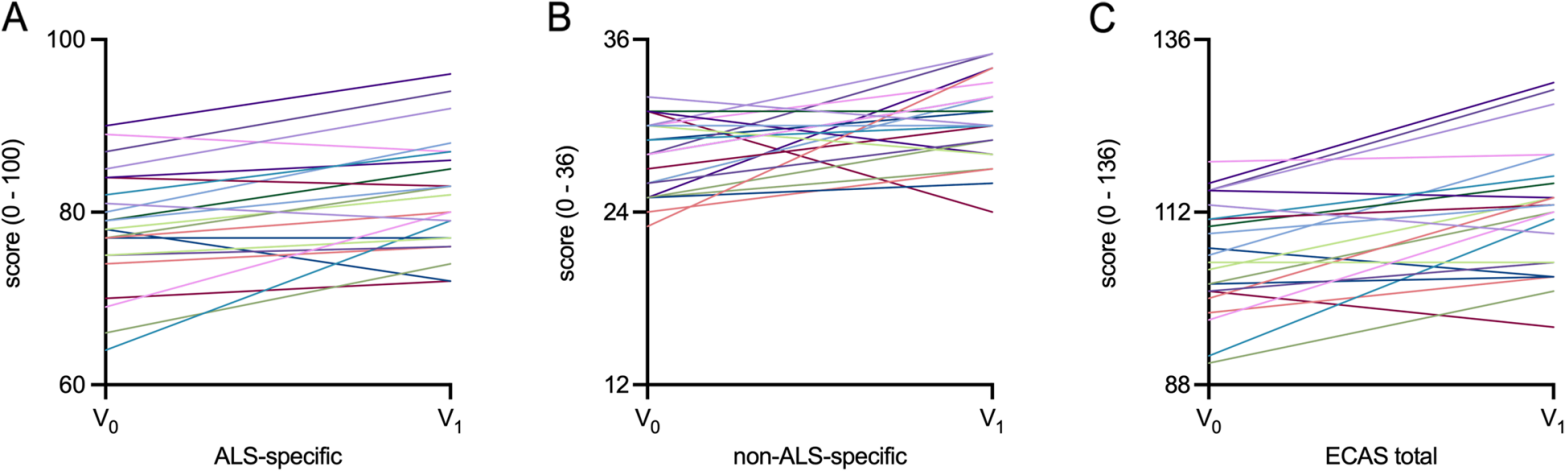
Spaghetti plots for longitudinal changes in ECAS of SMA cohort Spaghetti plots for **A**: ALS-specific subscore, **B**: non-ALS-specific subscore, **C**: ECAS total score. ALS: amyotrophic lateral sclerosis; ECAS: Edinburgh Cognitive and Behavioral ALS Screen; SMA: spinal muscular atrophy. V0: Baseline visit before treatment initiation; V1: Follow-up visit 14 months after treatment initiation. Each line represents an individual patient

**Fig. 4:**
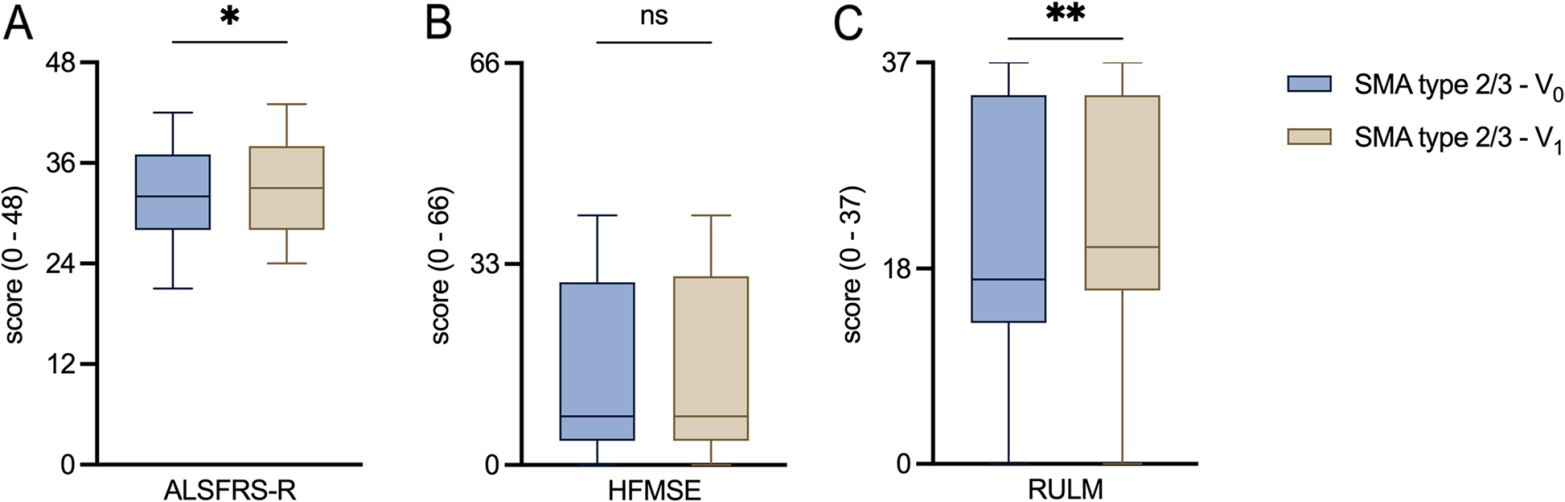
Comparison of motor outcome scores before and after treatment initiation **A**, **B**, **C**: Box plots of the motor outcome scores before and after treatment initiation with nusinersen in SMA cohort. ALSFRS-R: ALS Functional Rating Scale Revised; HFMSE: Hammersmith Functional Motor Scale Expanded; RULM: Revised Upper Limb Module; SMA: Spinal muscular atrophy V0: Baseline visit before treatment initiation; V1: Follow-up visit 14 months after treatment initiation. Box plots show median (horizontal line), inter-quartile range (boxes) and scores outside of inter-quartile range (whiskers). Calculated by Wilcoxon signed-rank test. Significance levels: * *p* < 0.05; ** *p* < 0.01; ns: statistically not significant

**Fig. 5:**
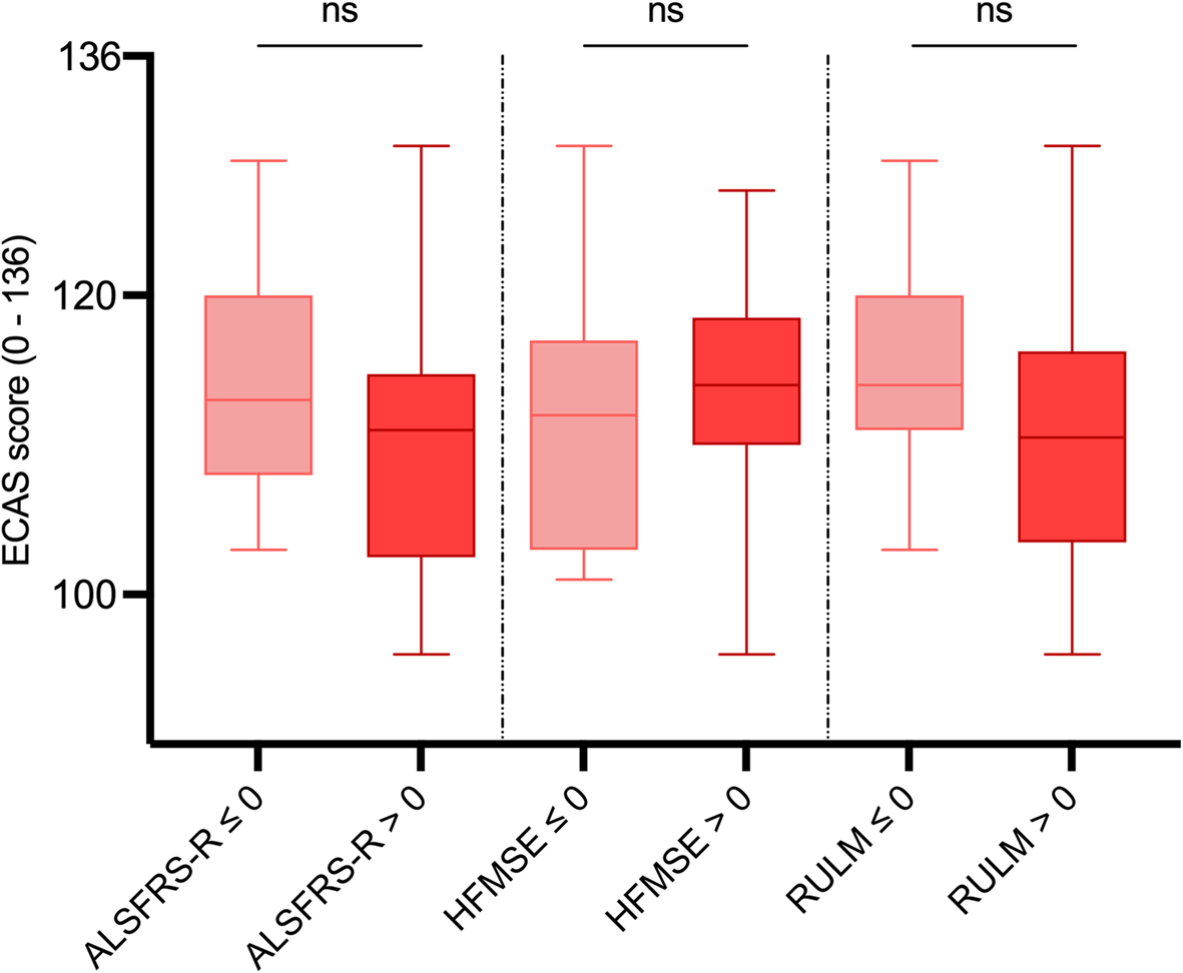
Comparison of ECAS total scores between SMA type 2/3 patients with improvement (score change > 0) and without improvement (score change ≤ 0) in motor outcome scores. ALSFRS-R: ALS Functional Rating Scale Revised; ECAS: Edinburgh Cognitive and Behavioral ALS Screen; HFMSE: Hammersmith Functional Motor Scale Expanded; RULM: Revised Upper Limb Module; ns: statistically not significant; Box plots show median (horizontal line), inter-quartile range (boxes) and scores outside of inter-quartile range (whiskers). Calculated by Mann–Whitney U-test

The original article [1] has been updated.
