# Supplementary material for: Cognitive performance of adult patients with SMA before and after treatment initiation with nusinersen
Source: BMC Neurol. 2023 Jun 6;23:216. doi: 10.1186/s12883-023-03261-z (PMC10243018; doi:10.1186/s12883-023-03261-z)
Supplement: Supplementary file 1 — Supplementary Material 1 [file 12883_2023_3261_MOESM1_ESM.docx]

**Table S1 - Partial correlation analysis for ECAS scores and motor function scores before and after treatment initiation**

| **ECAS outcome scores** | **ALSFRS-R** | | **HFMSE** | | **RULM** | |
| --- | --- | --- | --- | --- | --- | --- |
|  | ***p*** | ***r*** | ***p*** | ***r*** | ***p*** | ***r*** |
| **Language (0 – 28)**  V_0_  V_1_ | 0.481  0.945 | –0.163  0.016 | 0.204  0.842 | –0.289  –0.046 | 0.885  0.454 | –0.034  0.173 |
| **Verbal Fluency (0 – 24)**  V_0_  V_1_ | 0.516  0.790 | 0.150  –0.062 | 0.593  0.737 | 0.124  –0.078 | 0.428  0.799 | 0.183  –0.059 |
| **Executive Function (0 – 48)**  V_0_  V_1_ | 0.281  0.807 | –0.247  –0.057 | 0.450  0.997 | –0.174  0.001 | 0.459  0.871 | –0.171  –0.038 |
| **Memory (0 – 24)**  V_0_  V_1_ | 0.546  0.569 | 0.140  0.132 | 0.564  0.806 | 0.134  0.057 | 0.276  0.658 | 0.249  0.103 |
| **Visuospatial (0 – 28)**  V_0_  V_1_ | 0.616  0.487 | –0.116  0.161 | 0.372  0.594 | –0.205  0.123 | 0.447  0.304 | –0.175  0.235 |
| **ALS-specific (0 – 100)**  V_0_  V_1_ | 0.641  0.626 | –0.108  –0.113 | 0.697  0.561 | –0.090  –0.135 | 0.963  0.667 | –0.011  –0.100 |
| **Non-ALS-specific (0 – 36)**  V_0_  V_1_ | 0.561  0.494 | 0.135  0.158 | 0.609  0.713 | 0.119  0.085 | 0.287  0.494 | 0.244  0.158 |
| **ECAS total (0 – 136)**  V_0_  V_1_ | 0.827  0.905 | –0.051  –0.028 | 0.876  0.768 | –0.036  –0.069 | 0.748  0.938 | 0.074  –0.018 |

ALSFRS-R: ALS functional rating scale-revised; ECAS: Edinburgh Cognitive and Behavioral ALS Screen; HFMSE: Hammersmith Functional Motor Scale Expanded; RULM: Revised Upper Limb Module; V_0_: Baseline visit before treatment initiation; V_1_: Follow-up visit 14 months after treatment initiation; *p* < 0.05 considered statistically significant; *r*: Pearson coefficient controlling for the effect of age and education
